# Evidence-based intrapartum practice and its associated factors at a tertiary teaching hospital in the Philippines, a descriptive mixed-methods study

**DOI:** 10.1186/s12884-020-2778-5

**Published:** 2020-02-05

**Authors:** Chisato Masuda, Shirley Kristine Ferolin, Ken Masuda, Chris Smith, Mitsuaki Matsui

**Affiliations:** 1 Department of Global Health, Nagasaki University School of Tropical Medicine and Global Health, Sakamoto 1-12-4, Nagasaki, 852-8523 Japan; 2 Department of Obstetrics and Gynaecology, Southern Philippines Medical Centre, JP Laurel Avenue, Bajada, Davao City, 8000 The Philippines; 3 Department of Clinical Research, London School of Hygiene and Tropical Medicine, Keppel Street, London, WC1E7HT UK

**Keywords:** Evidence based practice (MeSH terms), Intrapartum care, Second stage labour (MeSH terms), Third stage labour (MeSH terms), Episiotomy (MeSH terms), Fundal pressure, Obstetric anal sphincter injuries

## Abstract

**Background:**

Evidenced-based practice is a key component of quality care. However, studies in the Philippines have identified gaps between evidence and actual maternity practices. This study aims to describe the practice of evidence-based intrapartum care and its associated factors, as well as exploring the perceptions of healthcare providers in a tertiary hospital in the Philippines.

**Methods:**

A mixed-methods study was conducted, which consisted of direct observation of intrapartum practices during the second and third stages, as well as semi-structured interviews and focus group discussions with care providers to determine their perceptions and reasoning behind decisions to perform episiotomy or fundal pressure. Univariate and multivariate logistic regression were used to analyse the relationship between observed practices and maternal, neonatal, and environmental factors. Qualitative data were parsed and categorised to identify themes related to the decision-making process.

**Results:**

A total of 170 deliveries were included. Recommended care, such as prophylactic use of oxytocin and controlled cord traction in the third stage, were applied in almost all the cases. However, harmful practices were also observed, such as intramuscular or intravenous oxytocin use in the second stage (14%) and lack of foetal heart rate monitoring (57%). Of primiparae, 92% received episiotomy and 31% of all deliveries received fundal pressure. Factors associated with the implementation of episiotomy included primipara (adjusted Odds Ratio [aOR] 62.3), duration of the second stage of more than 30 min (aOR 4.6), and assisted vaginal delivery (aOR 15.0). Factors associated with fundal pressure were primipara (aOR 3.0), augmentation with oxytocin (aOR 3.3), and assisted delivery (aOR 4.8). Healthcare providers believe that these practices can prevent laceration. The rate of obstetric anal sphincter injuries (OASIS) was 17%. Associated with OASIS were assisted delivery (aOR 6.0), baby weights of more than 3.5 kg (aOR 7.8), episiotomy (aOR 26.4), and fundal pressure (aOR 6.2).

**Conclusions:**

Our study found that potentially harmful practices are still conducted that contribute to the occurrence of OASIS. The perception of these practices is divergent with current evidence, and empirical knowledge has more influence. To improve practices the scientific evidence and its underlying basis should be understood among providers.

## Background

Quality of care is a focus area for improvement to reduce avoidable mortality and morbidity in mothers and newborn babies. According to the WHO Quality of Care Framework for maternal and newborn health, evidence-based practice is a key quality of care component [1]. Two entities underpin the implementation of evidence-based care; firstly, is the provision of ‘recommended’ practices, which have evidence of effectiveness and generally facilitate the physiological process of birth. The second is the avoidance of ‘not recommended’ practices, which are often invasive medical interventions, and have proved ineffective or harmful if provided in a routine manner. However, studies have identified gaps between recommended and actual practices [2]. Essential routine monitoring and assessment during labour as well as key practices are not sufficiently conducted, compounded by inappropriate infrastructures and supplies [3–5]. Mistreatment and abusive actions, including unnecessary interventions, are also common in health facility deliveries [4, 6].

The Ministry of Health in the Philippines adopted a policy on Essential Intrapartum and Newborn Care (EINC) in 2009 [7]. The vital part of the policy is the implementation of evidence-based practices, which consist of recommended practices during the intrapartum period. Recommended practices for newborn care are time-bound interventions at the time of birth and elimination of unnecessary interventions. Over 14,000 health workers in 252 hospitals have been trained since the end of 2015 [8]. Whereas this country-wide effort resulted in an improvement of newborn care practices, inappropriate maternal care practices persisted at tertiary level hospitals according to an evaluation of EINC practices [9]. The effectiveness of didactic training approaches for maternal care were questioned in this report; however, the reasons and context behind the poor compliance with guidelines were not well explored. Therefore, this study aims to describe the practice of evidence-based intrapartum care and its associated factors, as well as exploring the perception of healthcare providers in a tertiary teaching hospital in the Philippines.

## Methods

### Study design

This study was a mixed-methods study with a convergent parallel design. Quantitative and qualitative data were concurrently collected and then merged later for analysis.

### Study setting

This study was conducted at a maternity unit in the Southern Philippines Medical Centre in Davao City, the Philippines. This medical centre manages both low- and high-risk pregnancy cases and accepts referrals across Mindanao Island. There were 16,054 deliveries in 2017, including 11,292 normal spontaneous vaginal deliveries. This hospital also has an educational function for the training of medical, nursing, and midwifery students, as well as staff from primary and secondary healthcare facilities.

### Study participants

Women were recruited to this study upon entering the delivery room with singleton cephalic pregnancy and a vital foetus. The sample consisted of parturient women at the second stage of labour observed by the first author [CM] who attended sequential deliveries per order of admission, separated by resting periods. Epidural analgesia cases were excluded as they may have been associated with increased assisted vaginal delivery [10]. Emergency caesarean section cases were withdrawn. All mothers were informed before entering the delivery room that observation of care would be conducted during their delivery, as well as data collection from their medical records, and that all data would be treated anonymously. Women provided verbal consent and had the opportunity to opt out.

Healthcare providers (medical doctors, nurses, and midwives) who assisted deliveries at the maternity unit during the study period were invited. The study protocol was discussed and confirmed in staff meetings at the study site. Written consent to participate in the study was obtained from the health care providers.

### Data collection

#### Quantitative strand

Intrapartum practices by healthcare providers during the second and third stages of labour were directly observed between May 6th and June 9th, 2018. Observed practices were selected from the latest WHO recommendations on intrapartum care [11]. Five practices (duration of the second stage, birth position, method of pushing, episiotomy, and fundal pressure) out of six in the second stage, and the four recommended practices (prophylactic uterotonics, delayed umbilical cord clamping, controlled cord traction (CCT), and uterine massage) in the third stage of labour were evaluated. One practice in the second stage (techniques for preventing perineal trauma) was excluded from our observation, because it is not commonly taught and utilised in the study site. A list of the recommendations is attached in Additional file 4: Annex 1. In addition, the frequency and method of foetal heart rate (FHR) monitoring and application of labour augmentation were observed. Medical records were reviewed to systematically collect the following information on the parturient and the newborn baby: parity, age, gestational week, fundal height on admission, complication during current pregnancy, past medical history, mode of delivery, degree of perineal laceration, and baby weight and condition at birth. We calculated the sample size with an assumption that the episiotomy rates in primiparae and multiparae were 80 and 25%, respectively. With a 10% error range and 5% level of significance, 62 primiparae and 73 multiparae samples were required. Considering a 10% rate of missing data, we planned to observe 69 primiparous and 81 multiparous women.

#### Qualitative strand

Semi-structured interviews were conducted with health care providers to confirm the reason for either episiotomy or fundal pressure during observation. Interviews were conducted immediately after the delivery by posing the question, *What was the reasoning to conduct episiotomy and/or fundal pressure?* In addition, Focus Group Discussions (FGD) were conducted with selected healthcare providers to explore their experience and perceptions regarding episiotomy and fundal pressure. All FGD sessions were conducted after the completion of observations. During the FGDs the results of observations and interviews were shared with the participants together with the existing evidence for those practices. Questions were asked to discuss how they recognise the benefit and adverse effects of those practices and evidence behind the guidelines. Information from the participants reached saturation both in the interview and FGD sessions.

### Data analysis

#### Quantitative strand

Descriptive statistics were used to show the characteristics of the participants and the observed intrapartum care. Chi-square and the Mann-Whitney *U* test were used to compare proportions and continuous variables without a normal distribution. Univariate and multivariate logistic regression analyses were performed to identify maternal, foetal, and environmental factors associated with non-recommended care; namely, episiotomy and fundal pressure [11]. We selected these practices because they are potentially harmful when routinely applied to pregnant women and are frequently misused [12–15]. Explanatory variables were selected based on findings in the literature and frequent reasons to perform them reported in the qualitative strand in this study. Additional analysis was performed to explore the association between potential risk factors and the occurrence of obstetric anal sphincter injuries (OASIS), which include 3rd and 4th degree lacerations. Odds ratio with 95 % confidence intervals were calculated in the analyses. Statistical analyses were performed using STATA software version 14 (StataCorp LLC, Texas, USA).

#### Qualitative strand

Narrative data from the semi-structured interviews and FGDs were parsed and categorized into units of meaningful information [16]. These categories were then linked together to identify themes on the decision-making processes of medical providers [17]. No software was used for these steps. The qualitative data were merged with the quantitative results, then similarity or convergence between qualitative and quantitative data were examined for further interpretation of findings.

## Results

### Characteristics of mother and delivery

A total of 170 deliveries were observed out of 1090 eligible vaginal deliveries at the study site. During the study period, 25 medical doctors, 28 midwives, and 25 nurses were observed throughout their intrapartum practice out of 28 doctors, 31 midwives, and 27 nurses working in the ward. A comparison of the characteristics of the study participants (*n* = 170) and non-observed cases (*n* = 920) is shown in Table 1. The proportion of primiparous women was significantly higher in the observed group than in the non-observed group.

**Table 1:** Comparison of the characteristics of study participants and non-observed cases

Characteristics	Observed (*n* = 170)	Non-observed (*n* = 920)	*p*-value
Primipara	54.1%	44.2%	0.018
Age (median and IQR)	23 [19–28]	24 [20–30]	0.074
Assisted vaginal birth	7.7%	7.6%	0.99

The characteristics of the parturient women, delivery processes, and maternal and neonatal outcomes are shown in Table 2. The proportions of term deliveries and women without complications were 89 and 78%, respectively. Most mothers delivered spontaneously, while vacuum extraction or forceps were applied in 16 cases (9%). Deliveries during dayshifts (6 am to 6 pm) or night shifts (6 pm to 6 am) were almost equivalent. A midwife was the most common birth attendant for vaginal delivery. The prevalence of 3rd or 4th degree of perineal or vaginal lacerations (OASIS) was 17%. Twenty-eight babies (17%) required resuscitation or admission to a neonatal intensive care unit.

**Table 2:** Characteristic of parturient woman, delivery process, and maternal and neonatal outcomes (*N* = 170)

	Frequency	Percent
Maternal age		
15–19	43	25.3
20–29	97	57.1
30–39	25	14.7
40–45	5	2.9
(median) [IQR]	(23) [19–28]	
Parity		
Primipara	88	51.8
Multipara	82	48.2
Gestational week		
Less than 37 weeks	16	9.4
37 - 41 weeks	151	88.8
More than 42 weeks	3	1.8
Fundal height [*N* = 143]		
Less than 32 cm	100	69.9
32 cm or more	43	30.1
(median) [IQR]	(30) [29–32]	
Complication during current pregnancy		
None	133	78.2
Hypertensive disorders	25	14.7
Gestational diabetes	6	3.5
Others	6	3.5
Duration of the 2nd stage of labour		
30 min or less	115	67.6
More than 30 min	55	32.4
(median) [IQR]	(19) [9–35]	
Mode of delivery		
Normal vaginal	154	90.6
Vacuum extraction or forceps	16	9.4
Time of delivery		
Between 6 pm and 6 am (night shift)	82	48.2
Between 6 am and 6 pm (day shift)	88	51.8
Birth attendant		
Midwife	119	70.0
Medical doctor	49	28.8
Nurse	2	1.2
Perineal or vaginal laceration		
None	29	17.1
1st degree	31	18.2
2nd degree	81	47.6
3rd degree	20	11.8
4th degree	9	5.3
Baby weight at birth		
Less than 2500 g	14	8.2
2500–3499 g	137	80.6
3500–3999 g	18	10.6
4000 g or more	1	0.6
(mean) [SD]	(2940) [415]	
Baby condition at birth		
Resuscitation or/and admission to NICU	28	16.5
Intrapartum foetal death	1	0.6

### Description of intrapartum care during the 2nd and 3rd stage of labour

Table 3 presents a description of intrapartum care during the 2nd and 3rd stages of labour.

**Table 3:** Description of intrapartum care during the 2nd and 3rd stages of labour (*N* = 170)

	Frequency	Percent
Position at the birth of baby		
Supine	3	1.8
Semi-Fowler’s positions (less than 45°)	167	98.2
Method of pushing		
Not forced	126	74.1
Valsalva manoeuvre instructed	44	25.9
Episiotomy		
Performed – median	98	57.6
Performed – medio-lateral	3	1.8
Not performed	69	40.6
Episiotomy by parity		
Performed in primiparae [*n* = 88]	81	92.0
Performed in multiparae [*n* = 82]	20	24.4
Fundal pressure		
Performed	53	31.2
Not performed	117	68.8
Foetal heart rate monitoring		
Not monitored	97	57.1
Intermittent auscultation	33	19.4
Cardiotocograph	40	23.5
Labour augmentation by oxytocin		
Not conducted	110	64.7
By drip infusion only	36	21.2
By injection (im or iv)	21	12.4
By drip infusion and injection (im or iv)	3	1.8
Prophylactic use of oxytocin in the third stage		
Administrated	170	100
Dose of oxytocin		
10 IU	149	87.7
Less than 10 IU	21	12.4
Delayed umbilical cord clamping [*n* = 138]		
Performed	69	50.0
Not performed	69	50.0
Controlled cord traction		
Performed	168	98.8
Not performed	2	1.2
Counter pressure during CCT [*n* = 168]		
Conducted	157	93.4
Not conducted	11	6.6

#### Position during the 2nd stage of labour

All mothers had either a semi-Fowler’s or a supine position at the birth of the baby.

#### Method of pushing

In 26% of births the Valsalva manoeuvre was applied to encourage mothers to keep pushing without breathing.

#### Episiotomy

Episiotomy was performed by the median method in 58% of all mothers, and in 2% by the medio-lateral method. Local anaesthesia for episiotomy was rarely used (for only one woman). The episiotomy rate was 92% in the primiparae subgroup.

#### Fundal pressure

Fundal pressure was performed in 31% of participating mothers. The fundal pressure manoeuvre involved the healthcare provider placing their forearm on the fundus and grasping the handle located on the side of the delivery bed with another hand, forming a “T-shape”, then applying pressure. The initiation of fundal pressure, within 30 min of full dilatation of the cervix or after admittance into the delivery room, was observed in 62% of observed cases.

#### Foetal heart rate monitoring

More than half of the mothers did not receive FHR monitoring during the 2nd stage. The median frequency and interval of the intermittent auscultation was once (IQR 1–2) and 19 min (IQR 13–32), respectively.

#### Labour augmentation by oxytocin

Intramuscular or intravenous oxytocin injection was performed in 24 women. Observation of uterine contraction was not conducted during or immediately after the injection.

#### Prophylactic use of oxytocin during the third stage of labour

All 170 cases received intramuscular injection of oxytocin. However, 21 cases (12%) did not receive the full defined dose (10 IU) as recommended in the national guideline, since two to five units of oxytocin were injected for labour augmentation during the 2nd stage.

#### Delayed umbilical cord clamping

Delayed umbilical cord clamping is recommended in the national guideline when the baby does not require resuscitation. However, it was applied in only 50% of deliveries out of 138 cases observed.

#### Controlled cord traction

Most of the placental deliveries were conducted using CCT (99%). Suprapubic counter pressure was applied in 93% of the CCT cases.

#### Uterine massage

After delivery of the placenta, uterine massage was performed in 11 women (7%).

### Perception of potentially harmful practices and the evidence behind the guidelines

We conducted semi-structured interviews with 16 medical doctors, 19 midwives, and 4 nurses. We recruited healthcare providers each time episiotomy or fundal pressure was observed. For FGDs, six doctors, five nurses, and six midwives participated. The participants were selected using convenience sampling based on their availability. Three sessions were organised separately for medical doctors, midwives, and nurses. Each FGD lasted about 1.5 h.

Interviews and FGDs with healthcare providers explored their understanding and perceptions of conducting potentially harmful practices.

#### Perception of the episiotomy in primiparae

Healthcare providers reported that primiparae without episiotomy were at risk of OASIS due to the characteristic of their vagina and perineum, such as *“small”*, *“not elastic”*, *“contracted”*, and *“tight”*; and that episiotomy was a protective measure against severe, zigzag, or multiple laceration. Some doctors and midwives also said that such laceration is *“difficult to suture”* and *“takes time to repair”*, while they were tending to many deliveries. *“Large baby”* was one of the reasons to perform episiotomy. Providers assessed the size of the baby by the fundal height; however, the evaluation of foetal macrosomia differed by person, ranging from 28 cm to 32 cm. Providers reported recognising negative effects of episiotomy, such as *“infection”*, *“pain”*, and *“blood loss”*.

#### Perception of fundal pressure

Although all healthcare providers knew that fundal pressure is not recommended in the national guidelines, from their experiences they believed that it was effective to *“help the baby’s head descending”*, *“accelerate the 2*^*nd*^ *stage”*, or *“hasten the delivery”.* Reported reasons for performing fundal pressure included *“foetal head descending is not improving”*; *“long or prolonged 2*^*nd*^ *stage”*; and *“weak maternal pushing and maternal effort failed”*, described as *“mother stopped pushing in a few seconds”*. Because of trust in its effectiveness, fundal pressure was often selected as the first option to hasten the second stage of labour to avoid vacuum extraction or caesarean section. Healthcare providers reported that the equipment for vacuum extraction is single-use and costly, and its avoidance reduces out-of-pocket payment for the patient. They also mentioned that emergency caesarean section is often difficult because of the lack of operation room availability. Healthcare providers reported recognizing the negative effects of fundal pressure such as *“pain”*, *“uterine rupture”*, and *“hematoma or bruise of abdomen”*.

#### Long duration of the 2nd stage

*“Long or prolonged 2*^*nd*^ *stage”* was one of the reasons to apply fundal pressure, and a *“long duration”* was described from 30 min to two hours for primiparae, and 30 min to one hour for multiparae.

### Factors associated with healthcare providers performing potentially harmful practices

Additional file 1: Table S1 and Additional file 2: Table S2 show the results of bivariate and multivariable analyses on the relationships between maternal, foetal, and environmental factors with episiotomy and fundal pressure, respectively. We arbitrarily selected these explanatory variables in the multiple logistic regression model separately for episiotomy and fundal pressure. The number of explanatory variables were limited to six in episiotomy and five in fundal pressure based on the number of women receiving those practices.

Factors associated with episiotomy were primipara (adjusted odds ratio [95% confidence interval]: aOR 62.3 [16.3–237.1]), more than 30 min duration of the second stage (aOR 4.6 [1.2–17.7]), and assisted vaginal delivery by vacuum extraction or forceps (aOR 15.0 [1.2–192.0]). Having maternal complications was negatively associated with performing episiotomy (aOR 0.10 [0.02–0.45]).

Factors associated with implementation of fundal pressure were primipara (aOR 3.0 [1.4–6.7]), labour augmentation by oxytocin (aOR 3.3 [1.5–7.0]), and assisted vaginal delivery (aOR 4.8 [1.3–18.0]).

We omitted ‘Birth Attendant’ from the multiple regression analyses, because of collinearity between the birth attendant and mode of delivery. Instrumental delivery, such as vacuum extraction, is usually positively associated with practices of episiotomy and fundal pressure. Therefore, mode of delivery was included a priori in our analysis. However, vacuum extraction and forceps delivery can be performed only by medical doctors in the study site. If we included birth attendant, which was categorized as ‘medical doctor’ or ‘midwife or nurse’, in the model, it automatically produced ‘zero cell’, weakening the validity of the analyses.

### Associated factors for OASIS

Bivariate and multivariate analyses were performed on the relationships between maternal, foetal, and care-related factors and OASIS (Additional file 3: Table S3). Although parity, duration of the second stage, and labour augmentation by oxytocin have significant relationships with the occurrence of OASIS in the univariate analysis, these factors were omitted in the multivariate model because of their collinearity with the Valsalva manoeuvre (method of pushing), episiotomy, and fundal pressure. Assisted vaginal delivery (aOR 6.0 [1.6–22.4]), baby weight of more than 3.5 kg (aOR 7.8 [1.7–36.6]), episiotomy (aOR 26.4 [2.3–299.0]), and fundal pressure (aOR 6.2 [2.1–18.2]) were positively associated with OASIS.

## Discussion

This study used international evidence-based guidelines to evaluate the quality of intrapartum care in a tertiary teaching hospital in the Philippines. We found that active management of the third stage of labour using oxytocin and CCT with counter pressure was conducted in the majority of deliveries. It has been shown that some practices which are potentially harmful to mother and foetus need to be changed; specifically, FHR monitoring (absent in 57% and insufficient in 19% of cases), augmentation with oxytocin (14% by injection and 21% in drip infusion without monitoring), episiotomy (in 92% of primiparae), and fundal pressure (in 31% of cases).

The reasons for potentially harmful practices such as systematic episiotomy in primiparae, and frequent use of fundal pressure was derived from the local culture of the health care providers. They believe that these are good practices to protect the perineum or to facilitate the delivery process. In the following section we discuss the practices that should change.

### Lack of FHR monitoring

FHR monitoring is an essential intrapartum practice to detect signs of hypoxaemia and acidosis. Since frequent and intense uterine contraction is common during the second stage, it is recommended that FHR monitoring be conducted every five minutes by intermittent auscultation [18]. However, more than half of the cases were not monitored, and intermittent auscultation was applied in only 19% of the cases with an average interval of auscultation of 19 min. The risk of stillbirth was shown to be four to seven times higher when FHR was not monitored at least every hour during the 1st and 2nd stages of labour, in a study at a tertiary hospital in Nepal [19]. This study indicates that healthcare workers systematically miss the opportunity to detect foetal asphyxia. This might have been a contributing factor to intrapartum foetal death and the 28 newborn resuscitations and NICU admissions. A possible reason for this malpractice is that the national guideline has no clear recommendation on the frequency of intermittent auscultation [20]. These findings indicate that the lack of FHR monitoring should be improved as soon as possible, and the national guidelines should make a clear recommendation on the method of monitoring and evaluation of FHR with necessary actions in cases of abnormality. A nationwide investigation is also recommended to assess the frequency of FHR monitoring and the reasons for not monitoring FHR.

### Improper use of oxytocin during the 2nd stage of labour

This study found that one in three women received augmentation of labour without appropriate monitoring. Use of oxytocin prior to confirmation of delay in labour may increase the risk of uterine hyperstimulation, tachysystole, and foetal heart rate alterations [21]. Because its effects cannot be controlled, intramuscular or intravenous bolus administration of oxytocin increases the risk of uterine rupture, severe foetal asphyxia, and foetal death. In this study, however, 24 women (14%) received oxytocin. This practice should be immediately abandoned and strongly discouraged by the national guidelines.

### Episiotomy

We found that episiotomy was routinely provided to primiparae, contrary to the WHO recommendation which states that an *“acceptable*” *rate of episiotomy is difficult to determine*, and the national policy of selective episiotomy which is defined as *no episiotomy unless it is necessary for maternal or foetal reasons* [20]. The guideline is supported by studies showing that routine episiotomy is not effective to reduce vaginal and perineal lacerations regardless of the parity [12, 13]. This contrasts with the perceptions of providers in this study, who believe that primiparae have a higher risk of OASIS without episiotomy due to the rigidity of their perineum. There are similar findings in studies in Oman, Cambodia, and Vietnam [22–24]. Other studies have shown that primiparity is the most common factor associated with the decision to provide episiotomy [23, 25–27]. Findings from previous and the present study indicate that healthcare providers conduct episiotomy based on their own experience and recognition rather than recommendations derived from scientific evidence. Our study has also shown that a second stage of labour of more than 30 min duration and application of assisted vaginal delivery were associated with an increase in episiotomy rate. According to the WHO guideline, in primipara up to three hours duration of the second stage is considered normal [11]. However, our findings indicate that healthcare providers conduct episiotomy much earlier than necessary to facilitate the delivery. This may be due to the request of the mother to end the labour pain as soon as possible, or environmental constraints such as shortage of providers or limited number of delivery beds [11]. Assisted vaginal birth facilitates rapid descent of the foetal head and insertion of equipment extends the vaginal canal, thus mechanically contributing to an increased probability of OASIS. As shown in our qualitative investigation, healthcare providers believed that episiotomy itself is a preventive measure for laceration. Therefore, an increase in the episiotomy rate can be explained by perception, especially when instrumental delivery is conducted.Both fundal pressure and FHR monitoring did not show association with episiotomy after controlling for potential confounding factors. Episiotomy can be applied when foetal asphyxia is suspected, detectable only by FHR monitoring. However, our results suggest that neither fundal height nor FHR monitoring were a source of decision-making regarding the practice of episiotomy.In addition, it should be highlighted that few providers used local anaesthesia for episiotomy, thus deteriorating the quality of care. Provision of effective and sufficient anaesthesia is an essential procedure to reduce unnecessary pain and quell anxiety provoked by interventions.

### Fundal pressure

Our study found that fundal pressure was applied in 31% of observed cases, and that it was dominantly performed in primiparous women (43%). Other associated factors were labour augmentation by oxytocin and assisted vaginal birth. Providers reported that it has been shown that fundal pressure is effective to hasten the 2nd stage of labour. These qualitative findings explained our quantitative findings; specifically, that providers applied fundal pressure to accelerate the delivery and to avoid operative delivery. Contrary to their perceptions, fundal pressure is strongly not recommended in Philippines national guidelines [20], since it does not change desirable maternal outcomes such as duration of the 2nd stage, instrumental delivery, or caesarean section, as well as neonatal outcomes such as low arterial cord pH and Apgar scores [14]. Fundal pressure may also increase the occurrence of severe laceration and cervical tears, and the possibility of uterine rupture [15, 28, 29]. Excessive fundal pressure is described by mothers as painful, forceful, and even an abusive experience [30]. These findings also indicate that providers should be aware of the established evidence behind the recommendation and the possible harmful effects of fundal pressure.

Apart from the perception of healthcare providers, our study identified structural reasons for them to perform fundal pressure. The first reason is financial constraints. The Philippine Health Agenda for 2016 to 2022 envisages a universal healthcare system to protect underprivileged people from the high cost of medical services [31]. However, the cost for vacuum extraction is 3000 Philippines pesos (US $56), which is not reimbursed to the patient from health insurance. Once the providers learn that the parturient is poor but needs an intervention to facilitate the birth process, their first choice is fundal pressure because there might be no payment for consumables or equipment. Approximately 93% of the population were covered by the National Health Insurance Program in 2017 [32]. Vacuum extraction is an important component of basic emergency obstetric and neonatal care; therefore, it is recommended to include it in the insurance system to discourage application of fundal pressure.

### Obstetric anal and sphincter injuries

Risk factors for OASIS include primiparity, gestational diabetes, macrosomia, malpresentation or malposition of the foetus, assisted vaginal delivery, and episiotomy. A sub-analysis for primipara and non-instrumental deliveries in a systematic review of randomised controlled trials reported that OASIS prevalence was between 0 and 16% (average 3%) in a restrictive episiotomy group; and between 0 and 14% (average 5%) in a liberal use of episiotomy group [13]. National aggregated data from twenty European countries showed that the OASIS rates were between 0.1% in Romania to 5% in Iceland [33]. It is difficult to determine the *standard* prevalence of OASIS at a facility level since parturient characteristics differ for each health facility. However, our study has shown that OASIS prevalence among primiparae was 28%, which is much higher than previous findings. This study confirmed that birthweights greater than 3.5 kg, episiotomy, fundal pressure, and instrumental delivery were significantly associated with the occurrence of OASIS, consistent with the literature [34–36]. OASIS has both short- and long-term severe consequences, such as pain, infection, dyspareunia, sexual dysfunction, and anal incontinence [37, 38]. Therefore, minimising risk factors is important to avoid OASIS. The rate of instrumental deliveries in our observed cases was 9% (16/170). However, in 13 cases (81%) it was applied within one hour in the second stage of labour. Since FHR was not appropriately monitored, careful observation of maternal and foetal conditions may contribute to reduce the application of instrumental deliveries. Application of episiotomy should be improved and not routinely conducted to primiparae. It has been suggested that medio-lateral episiotomy is safer than median incision [11]. Median episiotomy is a known risk factor for OASIS, especially in operative deliveries, whereas medio-lateral or lateral episiotomy has a protective effect [39–41]. Fundal pressure should be avoided because of its harmfulness. Another key issue would be to carefully convey to pregnant women the risk factors and respectful midwifery care throughout pregnancy and delivery. It has been reported that the OASIS rate among primiparae in midwife-led birth centres was 0.2% in Japan [42, 43]. Midwives in Japan are not legally allowed to carry out invasive medical procedures, including episiotomy. Therefore, they only work with low-risk cases. They commit themselves to practicing evidence-based and humanized care during pregnancy and birth [44, 45]. These factors may contribute to reduce the risk of OASIS.

### Limitation and strength

This study has several limitations. First, there was selection bias of mothers at the sampling stage. Primiparous women were dominant in the observed group. Since observation started when a woman came into the delivery room with a diagnosis of the second stage of labour, we systematically missed cases with immediate delivery, which is more common in multiparae. However, this bias would not affect the relationship between maternal, foetal, and environmental factors and medical interventions or risk factors for OASIS.

Second, the potential for the Hawthorne effect could not be avoided due to the presence of an observer. The behaviour of healthcare providers may have positively improved knowing that their practices were being observed. Therefore, the observed performance of recommended practices may be higher, and potentially harmful practices may be lower than in reality [46]. However, if the observed practices can be considered as the best performance, this indicates there are still several problems regarding quality of care in the delivery room.

Thirdly, we did not consider the differences among individuals or types of providers. Episiotomy rates can vary considerably within the same group of providers in the same institution [47]. This study cannot draw conclusions on the effect disaggregated by individual or type of healthcare provider.

The strength of this study was the prospective data collection of clinical practice by direct observation with concurrent interviews with healthcare providers. Most previous studies on episiotomy and fundal pressure were conducted retrospectively. The direct observation method allowed us to describe details of the intrapartum practice and to accurately measure the performance rate of intrapartum care compared with self-reported measurement [48].

## Conclusion

Our study found four significant gaps between intrapartum practice and recommended evidence-based guidelines: a lack of FHR monitoring, improper use of oxytocin during labour, excessive use of episiotomy for primiparae, and application of fundal pressure. Our qualitative investigation has revealed that these unreasonable practices were derived from empirical knowledge and belief. Merely disseminating guidelines and recommendations is unlikely to improve practices, as quality of care will not be ensured. Scientific evidence and its underlying anatomy, physiology, and pathology should be well understood among providers. It is particularly important for a teaching hospital to apply national standards, since its practices are reproduced as the best practices for professionals at multiple levels of health facilities. Therefore, a continuous training mechanism with relevant monitoring and supervision should be mandated to ensure quality practices.

## Supplementary information

**Additional file 1: Table S1.** Relationship between maternal, foetal, and environmental factors and the performance of episiotomy.

**Additional file 2: Table S2.** Relationship between maternal, foetal, and environmental factors and the performance of fundal pressure.

**Additional file 3: Table S3.** Relationship between maternal, foetal and care-related factors and obstetric anal and sphincter injuries.

**Additional file 4: Annex 1.** WHO recommendations of care during labour and selection of observed practices in this study.

## Data Availability

The datasets generated and used for this study are available from the corresponding author on reasonable request.

## Abbreviations

CCT: Controlled cord traction
EINC: Essential intrapartum and newborn care
FGD: Focus Group Discussion
FHR: Foetal heart rate
OASIS: Obstetric anal sphincter injuries
PPH: Post-partum haemorrhage

## References

[1] Tunçalp Ö, Were WM, MacLennan C, Oladapo OT, Gülmezoglu AM, Bahl R (2015). Quality of care for pregnant women and newborns-the WHO vision. BJOG.

[2] Miller S, Abalos E, Chamillard M, Ciapponi A, Colaci D, Comande D (2016). Beyond too little, too late and too much, too soon: a pathway towards evidence-based, respectful maternity care worldwide. Lancet.

[3] Duysburgh E, Zhang WH, Ye M, Williams A, Massawe S, Sie A (2013). Quality of antenatal and childbirth care in selected rural health facilities in Burkina Faso, Ghana and Tanzania: similar finding. Tropical Med Int Health.

[4] Sharma G, Powell-Jackson T, Haldar K, Bradley J, Filippi V (2017). Quality of routine essential care during childbirth: clinical observations of uncomplicated births in Uttar Pradesh, India. Bull World Health Organ.

[5] Fisseha G, Berhane Y, Worku A, Terefe W (2017). Quality of the delivery services in health facilities in northern Ethiopia. BMC Health Serv Res.

[6] Kruk Margaret E, Kujawski Stephanie, Mbaruku Godfrey, Ramsey Kate, Moyo Wema, Freedman Lynn P (2014). Disrespectful and abusive treatment during facility delivery in Tanzania: a facility and community survey. Health Policy and Planning.

[7] World Health Organization Representative Office Philippines. Essential Intrapartum and Newborn Care (EINC) Evidence-based Standard Practices. http://origin.wpro.who.int/philippines/areas/maternal_child_nutrition/newborn_mother_care/einc_protocols/en/. Accessed 14 April 2018.

[8] World Health Organization (2018). Second biennial progress report: 2016-2017 (Action Plan for Health Newborn Infants in the Western Pacific Region: 2014-2020).

[9] Silvestre MAA, Mannava P, Corsino MA, Capili DS, Calibo AP, Tan CF (2018). Improving immediate newborn care practices in Philippine hospitals: impact of a national quality of care initiative 2008-2015. Int J Qual Health Care.

[10] Anim-Somuah M, Smyth RM, Cyna AM, Cuthbert A (2018). Epidural versus non-epidural or no analgesia for pain management in labour. Cochrane Database Systematic Rev.

[11] World Health Organization (2018). WHO recommendations: intrapartum care for a positive childbirth experience.

[12] Carroli G, Mignini L (2009). Episiotomy for vaginal birth. Cochrane Database Systematic Rev.

[13] Jiang H, Qian X, Carroli G, Garner P (2017). Selective versus routine use of episiotomy for vaginal birth. Cochrane Database Systematic Rev.

[14] Hofmeyr GJ, Vogel JP, Cuthbert A, Singata M (2017). Fundal pressure during the second stage of labour. Cochrane Satabase Syst Rev.

[15] Sturzenegger K, Schaffer L, Zimmermann R, Haslinger C (2017). Risk factors of uterine rupture with a special interest to uterine fundal pressure. J Perinat Med.

[16] Teddlie C, Tashakkori A (2009). Foundations of mixed methods research : integrating quantitative and qualitative approaches in the social and behavioral sciences.

[17] Bernard HR (2006). Research methods in anthropology : qualitative and quantitative approaches. (4th ed).

[18] Lewis D, Downe S, FIGO Intrapartum Fetal Monitoring Expert Consensus Panel (2015). FIGO consensus guidelines on intrapartum fetal monitoring: Intermittent auscultation. Int J Gynaecol Obstet.

[19] Kc A, Wrammert J, Clark RB, Ewald U, Malqvist M (2016). Inadequate fetal heart rate monitoring and poor use of partogram associated with intrapartum stillbirth: a case-referent study in Nepal. BMC Pregnancy Childbirth.

[20] Department of Health, Philippine Obstetrical and Gynecological Society (2012). Clinical practice guidelines on intrapartum and immediate postpartum care.

[21] World Health Organization (2014). WHO recommendations for augmentation of labour.

[22] Al-Ghammari K, Al-Riyami Z, Al-Moqbali M, Al-Marjabi F, Al-Mahrouqi B, Al-Khatri A (2016). Predictors of routine episiotomy in primigravida women in Oman. Appl Nurs Res.

[23] Schantz C, Sim KL, Ly EM, Barennes H, Sudaroth S, Goyet S (2015). Reasons for routine episiotomy: a mixed-methods study in a large maternity hospital in Phnom Penh, Cambodia. Reprod Health Matters.

[24] Trinh AT, Roberts CL, Ampt AJ (2015). Knowledge, attitude and experience of episiotomy use among obstetricians and midwives in Viet Nam. BMC Pregnancy Childbirth.

[25] Saadia Z (2014). Rates and indicators for episiotomy in modern obstetrics - a study from Saudi Arabia. Mater Sociomed.

[26] Ballesteros-Meseguer C, Carrillo-Garcia C, Meseguer-de-Pedro M, Canteras-Jordana M, Martinez-Roche ME (2016). Episiotomy and its relationship to various clinical variables that influence its performance. Rev Lat Am Enfermagem.

[27] Hussein SA, Dahlen HG, Duff M, Schmied V (2016). The barriers and facilitators to evidence-based episiotomy practice in Jordan. Women birth.

[28] Matsuo K, Shiki Y, Yamasaki M, Shimoya K (2009). Use of uterine fundal pressure maneuver at vaginal delivery and risk of severe perineal laceration. Arch Gyneco Obstet.

[29] Moiety FM, Azzam AZ (2014). Fundal pressure during the second stage of labor in a tertiary obstetric center: a prospective analysis. J Obstet Gynaecol Res.

[30] Balde MD, Diallo BA, Bangoura A, Sall O, Soumah AM, Vogel JP (2017). Perceptions and experiences of the mistreatment of women during childbirth in health facilities in Guinea: a qualitative study with women and service providers. Reprod Health.

[31] Cabral E (2016). The Philippine health agenda for 2016 to 2022. Phil J Int Med.

[32] Philippines Health Insurance Corporation (2018). Annual report 2017.

[33] Blondel B, Alexander S, Bjarnadottir RI, Gissler M, Langhoff-Roos J, Novak-Antolic Z (2016). Variations in rates of severe perineal tears and episiotomies in 20 European countries: a study based on routine national data in euro-Peristat project. Acta Obstet Gynecol Scand.

[34] Hirayama F, Koyanagi A, Mori R, Zhang J, Souza JP, Gülmezoglu AM (2012). Prevalence and risk factors for third- and fourth-degree perineal lacerations during vaginal delivery: a multi-country study. BJOG.

[35] Pergialiotis V, Vlachos D, Protopapas A, Pappa K, Vlachos G (2014). Risk factors for severe perineal lacerations during childbirth. Int J Gynaecol Obstet.

[36] Mahgoub S, Piant H, Gaudineau A, Lefebvre F, Langer B, Koch A (2019). Risk factors for obstetric anal sphincter injuries (OASIS) and the role of episiotomy: a retrospective series of 496 cases. J Gyneco Obstet Hum Reprod.

[37] Keriakos R, Gopinath D (2015). Obstetric anal sphincter injuries. J Acute Dis.

[38] Preston HL, Fowler GE (2016). Risk factors for and management of obstetric anal sphincter injury. Obstet Gynaecol Reprod Med.

[39] Kudish B, Blackwell S, McNeeley SG, Bujold E, Kruger M, Hendrix SL (2006). Operative vaginal delivery and midline episiotomy: a bad combination for the perineum. Am J Obstet Gynecol.

[40] Räisänen SH, Vehviläinen-Julkunen K, Gissler M, Heinonen S (2009). Lateral episiotomy protects primiparous but not multiparous women from obstetric anal sphincter rupture. Acta Obstet Gynecol Scand.

[41] Lund NS, Persson LK, Jango H, Gommesen D, Westergaard HB (2016). Episiotomy in vacuum-assisted delivery affects the risk of obstetric anal sphincter injury: a systematic review and meta-analysis. Eur J of Obstet Gynecol Reprod Biol.

[42] Kataoka Y, Eto H, Iida M (2013). Outcomes of independent midwifery attended births in birth centres and home births: a retrospective cohort study in Japan. Midwifery.

[43] Suto M, Takehara K, Misago C, Matsui M (2015). Prevalence of Perineal lacerations in women giving birth at midwife-led birth centers in Japan: a retrospective descriptive study. J Midwifery Women Health.

[44] Misago C, Umenai T, Noguchi M, Mori T, Mori T (2000). Satisfying birthing experiences in Japan. Lancet.

[45] Behruzi R, Hatem M, Fraser W, Goulet L, Ii M, Misago C (2010). Facilitators and barriers in the humanization of childbirth practice in Japan. BMC Pregnancy Childbirth.

[46] Oswald D, Sherratt F, Smith S (2014). Handling the Hawthorne effect: the challanges surrongind a participant observer. Rev Soc Stud.

[47] Graham ID, Carroli G, Davies C, Medves JM (2005). Episiotomy rates around the world: an update. Birth.

[48] Kawulich BB (2005). Participant observation as a data collection method. Forum: Qualitat Soc Res.

