# Supplementary material for: Evidence-based intrapartum practice and its associated factors at a tertiary teaching hospital in the Philippines, a descriptive mixed-methods study
Source: BMC Pregnancy Childbirth. 2020 Feb 5;20:78. doi: 10.1186/s12884-020-2778-5 (PMC7003416; doi:10.1186/s12884-020-2778-5)
Supplement: Supplementary file 1 — Additional file 1: Table S1. Relationship between maternal, foetal, and environmental factors and the performance of episiotomy. [file 12884_2020_2778_MOESM1_ESM.docx]

Additional file 1: Table S1. Relationship between maternal, foetal, and environmental factors and the performance of episiotomy

| Factors |  | Episiotomy |  |  | Odds Ratio | 95% CI |  |  |  |  |  |
| --- | --- | --- | --- | --- | --- | --- | --- | --- | --- | --- | --- |
|  |  | Frequency | % |  | Crude |  | p-value |  | Adjusted |  | p-value |
| Parity |  |  |  |  |  |  |  |  |  |  |  |
| Primipara |  | 81/88 | 92.0 |  | 35.9 | 10.0-128.3 | <0.001 |  | 62.3 | 16.3-237.1 | <0.001 |
| Multipara |  | 20/82 | 24.2 |  | ref |  |  |  | ref |  |  |
| Gestational week |  |  |  |  |  |  |  |  |  |  |  |
| Less than 37 weeks |  | 10/16 | 62.5 |  | 1.2 | 0.39-3.3 | 0.79 |  |  |  |  |
| 37 weeks or more |  | 91/154 | 59.1 |  | ref |  |  |  |  |  |  |
| Fundal height [N=143] |  |  |  |  |  |  |  |  |  |  |  |
| Less than 32 cm |  | 61/100 | 61.0 |  | ref |  |  |  | ref |  |  |
| 32 cm or more |  | 24/43 | 55.8 |  | 0.80 | 0.39-1.7 | 0.56 |  | 0.70 | 0.22-2.2 | 0.54 |
| Complication during current pregnancy |  |  |  |  |  |  |  |  |  |  |  |
| None |  | 86/133 | 64.7 |  | ref |  |  |  | ref |  |  |
| HDP, GDM, others |  | 15/37 | 40.5 |  | 0.37 | 0.17-0.80 | 0.008 |  | 0.10 | 0.02-0.45 | 0.003 |
| Duration of the 2nd stage of labour |  |  |  |  |  |  |  |  |  |  |  |
| 30 min or less |  | 53/113 | 46.9 |  | ref |  |  |  | ref |  |  |
| More than 30 min |  | 48/57 | 84.2 |  | 6.0 | 2.6-14.2 | <0.001 |  | 4.6 | 1.2-17.7 | 0.03 |
| Foetal heart rate |  |  |  |  |  |  |  |  |  |  |  |
| Not monitored |  | 49/97 | 50.5 |  | ref |  |  |  | ref |  |  |
| Monitored |  | 52/73 | 71.2 |  | 2.4 | 1.3-4.7 | 0.007 |  | 0.82 | 0.24-2.8 | 0.76 |
| Labour augmentation by oxytocin |  |  |  |  |  |  |  |  |  |  |  |
| Not conducted |  | 61/110 | 55.5 |  | ref |  |  |  |  |  |  |
| Conducted |  | 40/60 | 66.7 |  | 1.6 | 0.82-3.1 | 0.16 |  |  |  |  |
| Mode of delivery |  |  |  |  |  |  |  |  |  |  |  |
| Normal vaginal |  | 87/154 | 56.5 |  | ref |  |  |  | ref |  |  |
| Vacuum extraction or forceps |  | 14/16 | 87.5 |  | 5.4 | 1.1-25.3 | 0.017 |  | 15.0 | 1.2-192.0 | 0.04 |
| Birth attendant |  |  |  |  |  |  |  |  |  |  |  |
| Midwife or nurse |  | 63/121 | 52.1 |  | ref |  |  |  |  |  |  |
| Medical doctor |  | 38/49 | 77.6 |  | 3.2 | 1.5-7.0 | 0.002 |  |  |  |  |
| Time of birth |  |  |  |  |  |  |  |  |  |  |  |
| Between 6 pm and 6 am |  | 47/82 | 57.3 |  | ref |  |  |  |  |  |  |
| Between 6 am and 6 pm |  | 54/88 | 61.4 |  | 1.2 | 0.63-2.2 | 0.59 |  |  |  |  |
