# Supplementary material for: Evidence-based intrapartum practice and its associated factors at a tertiary teaching hospital in the Philippines, a descriptive mixed-methods study
Source: BMC Pregnancy Childbirth. 2020 Feb 5;20:78. doi: 10.1186/s12884-020-2778-5 (PMC7003416; doi:10.1186/s12884-020-2778-5)
Supplement: Supplementary file 2 — Additional file 2: Table S2. Relationship between maternal, foetal, and environmental factors and the performance of fundal pressure. [file 12884_2020_2778_MOESM2_ESM.docx]

Additional file 2: Table S2. Relationship between maternal, foetal, and environmental factors and the performance of fundal pressure

| Factors |  | F/P |  |  | Odds ratio | 95% CI |  |  |  |  |  |
| --- | --- | --- | --- | --- | --- | --- | --- | --- | --- | --- | --- |
|  |  | Frequency | % |  | Crude |  | p-value |  | Adjusted |  | p-value |
| Parity |  |  |  |  |  |  |  |  |  |  |  |
| Primipara |  | 38/88 | 43.2 |  | 3.4 | 1.6-7.0 | <0.001 |  | 3.0 | 1.4-6.7 | 0.007 |
| Multipara |  | 15/82 | 18.3 |  | ref |  |  |  | ref |  |  |
| Gestational week |  |  |  |  |  |  |  |  |  |  |  |
| Less than 37 weeks |  | 1/16 | 6.3 |  | 0.13 | 0.02-1.1 | 0.03 |  |  |  |  |
| 37 weeks or more |  | 52/154 | 33.8 |  | ref |  |  |  |  |  |  |
| Fundal height [N=143] |  |  |  |  |  |  |  |  |  |  |  |
| Less than 32 cm |  | 27/100 | 27.0 |  | ref |  |  |  |  |  |  |
| 32 cm or more |  | 18/43 | 41.9 |  | 1.9 | 0.90-4.2 | 0.08 |  |  |  |  |
| Complication during current pregnancy |  |  |  |  |  |  |  |  |  |  |  |
| None |  | 38/133 | 28.6 |  | ref |  |  |  |  |  |  |
| HDP, GDM, others |  | 15/37 | 40.5 |  | 1.7 | 0.79-3.7 | 0.17 |  |  |  |  |
| Duration of the second stage of labour |  |  |  |  |  |  |  |  |  |  |  |
| 30 min or less |  | 23/113 | 20.4 |  | ref |  |  |  | ref |  |  |
| More than 30 min |  | 30/57 | 52.6 |  | 4.3 | 2.1-9.1 | <0.001 |  | 2.4 | 0.99-5.6 | 0.05 |
| Foetal heart rate |  |  |  |  |  |  |  |  |  |  |  |
| Not monitored |  | 24/97 | 24.7 |  | ref |  |  |  | ref |  |  |
| Monitored |  | 29/73 | 39.7 |  | 2.0 | 1.0-3.9 | 0.04 |  | 1.0 | 0.44-2.4 | 0.95 |
| Labour augmentation by oxytocin |  |  |  |  |  |  |  |  |  |  |  |
| Not conducted |  | 23/110 | 20.9 |  | ref |  |  |  | ref |  |  |
| Conducted |  | 30/60 | 50.0 |  | 3.8 | 1.8-7.8 | <0.001 |  | 3.3 | 1.5-7.0 | 0.002 |
| Mode of delivery |  |  |  |  |  |  |  |  |  |  |  |
| Normal vaginal |  | 41/154 | 26.6 |  | ref |  |  |  | ref |  |  |
| Vacuum extraction or forceps |  | 12/16 | 75.0 |  | 8.3 | 2.4-28.8 | <0.001 |  | 4.8 | 1.3-18.0 | 0.02 |
| Birth attendant |  |  |  |  |  |  |  |  |  |  |  |
| Midwife or nurse |  | 25/121 | 20.7 |  | ref |  |  |  |  |  |  |
| Medical doctor |  | 28/49 | 57.1 |  | 5.1 | 2.4-11.0 | <0.001 |  |  |  |  |
| Time of birth |  |  |  |  |  |  |  |  |  |  |  |
| Between 6 pm and 6 am |  | 21/82 | 25.6 |  | ref |  |  |  |  |  |  |
| Between 6 am and 6 pm |  | 32/88 | 36.4 |  | 1.7 | 0.85-3.2 | 0.13 |  |  |  |  |
