# Supplementary material for: Evidence-based intrapartum practice and its associated factors at a tertiary teaching hospital in the Philippines, a descriptive mixed-methods study
Source: BMC Pregnancy Childbirth. 2020 Feb 5;20:78. doi: 10.1186/s12884-020-2778-5 (PMC7003416; doi:10.1186/s12884-020-2778-5)
Supplement: Supplementary file 3 — Additional file 3: Table S3. Relationship between maternal, foetal and care-related factors and obstetric anal and sphincter injuries. [file 12884_2020_2778_MOESM3_ESM.docx]

Additional file 3: Table S3. Relationship between maternal, foetal and care-related factors and obstetric anal and sphincter injuries

| Factors |  | Laceration |  |  | Odds Ratio | 95%CI |  |  |  |  |  |
| --- | --- | --- | --- | --- | --- | --- | --- | --- | --- | --- | --- |
|  |  |  |  |  | Crude |  | p-value |  | Adjusted |  | p-value |
| Maternal age |  | Frequency | % |  |  |  |  |  |  |  |  |
| 15-19 |  | 10/43 | 23.3 |  | ref |  |  |  |  |  |  |
| 20-29 |  | 16/97 | 16.5 |  | 0.65 | 0.27-1.6 | 0.35 |  |  |  |  |
| 30-45 |  | 3/30 | 10.0 |  | 0.37 | 0.09-1.5 | 0.16 |  |  |  |  |
| Parity |  |  |  |  |  |  |  |  |  |  |  |
| Primipara |  | 25/88 | 28.4 |  | 7.7 | 2.4-24.9 | <0.001 |  |  |  |  |
| Multipara |  | 4/82 | 4.9 |  | ref |  |  |  |  |  |  |
| Duration of the second stage of labour |  |  |  |  |  |  |  |  |  |  |  |
| 30 min or less |  | 7/113 | 6.2 |  | ref |  |  |  |  |  |  |
| More than 30 min |  | 22/57 | 38.6 |  | 9.5 | 3.4-26.5 | <0.001 |  |  |  |  |
| Labour augmentation by oxytocin |  |  |  |  |  |  |  |  |  |  |  |
| Not conducted |  | 13/110 | 11.8 |  | ref |  |  |  |  |  |  |
| Conducted |  | 16/60 | 26.7 |  | 2.7 | 1.2-6.2 | 0.02 |  |  |  |  |
| Mode of delivery |  |  |  |  |  |  |  |  |  |  |  |
| Normal vaginal |  | 19/154 | 12.3 |  | ref |  |  |  | ref |  |  |
| Vacuum extraction or forceps |  | 10/16 | 62.5 |  | 11.8 | 3.5-40.1 | <0.001 |  | 6.0 | 1.6-22.4 | 0.007 |
| Birth weights |  |  |  |  |  |  |  |  |  |  |  |
| Less than 3500 g |  | 23/151 | 15.2 |  | ref |  |  |  | ref |  |  |
| 3500 g or more |  | 6/19 | 31.6 |  | 2.6 | 0.88-7.4 | 0.08 |  | 7.8 | 1.7-36.6 | 0.009 |
| Valsalva maneuver |  |  |  |  |  |  |  |  |  |  |  |
| Not conducted |  | 16/126 | 12.7 |  | ref |  |  |  |  |  |  |
| Conducted |  | 13/44 | 29.6 |  | 2.9 | 1.2-6.8 | 0.02 |  |  |  |  |
| Episiotomy |  |  |  |  |  |  |  |  |  |  |  |
| Not conducted |  | 1/69 | 1.5 |  | ref |  |  |  | ref |  |  |
| Conducted |  | 28/109 | 27.7 |  | 26.1 | 3.0-225.9 | <0.001 |  | 26.4 | 2.3-299.0 | 0.008 |
| Fundal pressure |  |  |  |  |  |  |  |  |  |  |  |
| Not conducted |  | 7/117 | 6.0 |  | ref |  |  |  | ref |  |  |
| Conducted |  | 22/53 | 41.5 |  | 11.2 | 3.9-31.8 | <0.001 |  | 6.2 | 2.1-18.2 | 0.001 |
