# Supplementary material for: Evidence-based intrapartum practice and its associated factors at a tertiary teaching hospital in the Philippines, a descriptive mixed-methods study
Source: BMC Pregnancy Childbirth. 2020 Feb 5;20:78. doi: 10.1186/s12884-020-2778-5 (PMC7003416; doi:10.1186/s12884-020-2778-5)
Supplement: Supplementary file 4 — Additional file 4: Annex 1. WHO recommendations of care during labour and selection of observed practices in this study. [file 12884_2020_2778_MOESM4_ESM.docx]

Additional file 4: Annex 1. WHO recommendations of care during labour and selection of observed practices in this study

| Stage of labour | Care option | Recommendation | Category of recommendation / observation |
| --- | --- | --- | --- |
| Second | Definition and duration of the second stage of labour | 33. The use of the following definition and duration of the second stage of labour is recommended for practice.  — The second stage is the period of time between full cervical dilatation and birth of the baby | Recommended and observed. |
|  | Birth position | 34. For women without epidural analgesia, encouraging the adoption of a birth position of the individual woman’s choice, including upright positions, is recommended. | Recommended and observed. |
|  | Method of pushing | 36. Women in the expulsive phase of the second stage of labour should be encouraged and supported to follow their own urge to push. | Recommended and observed. |
|  | Techniques for preventing perineal trauma | 38. For women in the second stage of labour, techniques to reduce perineal trauma and facilitate spontaneous birth (including perineal massage, warm compresses and a “hands on” guarding of the perineum) are recommended, based on a woman’s preferences and available options. | Recommended but not observed. Because this technique is not commonly taught and utilised. |
|  | Episiotomy policy | 39. Routine or liberal use of episiotomy is not recommended for women undergoing spontaneous vaginal birth. | Not recommended and observed. |
|  | Fundal pressure | 40. Application of manual fundal pressure to facilitate childbirth during the second stage of labour is not recommended. | Not recommended and observed. |
| Third | Prophylactic uterotonics | 1. The use of uterotonics for the prevention of postpartum haemorrhage (PPH) during the third stage of labour is recommended for all births. 2. Oxytocin (10 IU, IM/IV) is the recommended uterotonic drug for the prevention of postpartum haemorrhage (PPH). | Recommended and observed. |
|  | Delayed umbilical cord clamping | 44. Delayed umbilical cord clamping (not earlier than 1 minute after birth) is recommended for improved maternal and infant health and nutrition outcomes. | Recommended and observed. |
|  | Controlled cord traction (CCT) | 45. In settings where skilled birth attendants are available, controlled cord traction (CCT) is recommended for vaginal births if the care provider and the parturient woman regard a small reduction in blood loss and a small reduction in the duration of the third stage of labour as important. | Recommended and observed. |
|  | Uterine massage | 46. Sustained uterine massage is not recommended as an intervention to prevent postpartum haemorrhage (PPH) in women who have received prophylactic oxytocin. | Not recommended and observed. |
